# Non-use of healthcare services among persons with mobility impairments in Cofimvaba, South Africa

**DOI:** 10.4102/ajod.v12i0.1112

**Published:** 2023-01-27

**Authors:** Babalwa Tshaka, Surona Visagie, Lieketseng Y. Ned

**Affiliations:** 1 Centre for Disability and Rehabilitation Studies, Faculty of Medicine and Health Sciences, Stellenbosch University, Cape Town, South Africa

**Keywords:** Access, mobility impairments, disability, healthcare, challenges, South Africa

## Abstract

**Background:**

Access to primary health care is a fundamental right for all. However, persons with disabilities are experiencing difficulties when accessing healthcare because of various environmental and personal barriers which may lead to nonuse of such services.

**Objectives:**

This study aimed to identify the challenges leading to non-use of healthcare services among persons with mobility impairments in Cofimvaba.

**Method:**

A descriptive qualitative design using snowball sampling was implemented. Semistructured interviews were conducted in isiXhosa with five participants who stopped accessing healthcare, using a self-developed interview guide. Inductive thematic analysis was used to develop codes and themes from the data.

**Results:**

Study findings revealed major challenges experienced by persons with mobility impairments in accessing healthcare. These included inaccessible roads, geographic inaccessibility, financial accessibility and indirect cost of care, having little or not many health problems, physical infrastructure difficulties within facilities, and attitudinal barriers.

**Conclusion:**

The findings indicated that persons with disabilities are experiencing a combination of structural and environmental challenges which make them stop accessing healthcare.

**Contribution:**

The article shares insights on access challenges that influence non-use of the often-needed healthcare services within the context of rural areas.

## Introduction

Approximately 1 billion (15%) of the world’s population has some form of disability. Eighty percent of these people live in low- and middle-income countries (LMICs) (WHO 2011). Among these countries, a majority of the population live in poverty (WHO 2011). Therefore, LMICs carry a combined burden of poverty and disability. The link between poverty and disability is well documented (Hanass-Hancock & McKenzie 2017; Mitra et al. 2017; UN 2018; WHO 2011). On one hand, disability increases the risk of poverty because of inadequate opportunities, poor access and impairment-related costs. On the other hand, poverty also increases the risk of disability through living circumstances, occupational risks and poor access to services such as high-quality education and healthcare, among others (Hanass-Hancock & McKenzie 2017). South Africa, because of its apartheid history, remains a deeply unequal country, with the majority of its population living in poverty. About 55% (30.4 million) of South Africans live in poverty. The South African disability statistics based on census 2011 indicate that 7.5% of the population are persons with disabilities (Stats SA 2011).

Because of the country’s inequalities, public healthcare in the country remains inequitable, with inadequate resources for rehabilitation. Accessing healthcare is a human right which is recognised by the South African Constitution under Section 27. South Africa’s ratification and signatory status of the Convention on the Rights of Persons with Disabilities (CRPD) (UN 2006), an international human rights framework, has also played a role in advocating for the rights of persons with disabilities to quality healthcare. Yet various authors have revealed that access to healthcare remains an unmet need in South Africa, and this is worse for poor persons with disabilities in rural areas (Grut et al. 2012; Moodley & Rose 2015; Mutwali & Rose 2019; Vergunst et al. 2015, 2017; Visagie & Schneider 2014). The recent United Nations (UN) (2018) report similarly revealed that persons with disabilities are three times more likely to be unable to access healthcare when needed and have poorer health outcomes than their peers without disabilities (UN 2018).

Rehabilitation access as a component of healthcare services remains a challenge too, with more than 50% of persons with disabilities having an unmet need for rehabilitation services (UN 2018).

Visagie and Swartz (2016), as well as Magaqa, Ariana and Polack (2021), confirmed these rehabilitation challenges in their recent studies. The difficulties often experienced by persons with disabilities include service accessibility and/or inaccessible physical or built environment; receiving lower-quality services and added extra costs for healthcare services (Kuper & Heydt 2019); discrimination and social stigma (Hashemi et al. 2020; Mitchell et al. 2022); and knowledge, information and communication barriers (Hashemi et al. 2020; Mitchel et al. 2022).

Different scholars have argued that it is crucial for persons with disabilities to access healthcare as their peers do or even more so, because they can be vulnerable to poor health and might develop secondary complications (Kuper & Heydt 2019; Sherry 2014; United Nations 2018). As confirmed by the UN (2018:73), Sustainable Development Goal (SDG) 3 shows that persons with disabilities are at a greater risk of developing secondary complications, have poor health and need healthcare more than other people. These services may be for either general healthcare or services specifically related to their impairments (Kuper & Heydt 2019).

The studies described show that persons with disabilities have more healthcare needs than their peers without disabilities. Yet they are more vulnerable to inaccessible and low-quality healthcare than others. They also show that many factors play a role in access challenges.

Geographical, racial and socio-economic differences in health service provision intersect with disability to further shape the disparities experienced by persons with disabilities in South Africa.

South Africa has many persons with mobility impairments because of its high rates of violence and trauma. Another driver of the prevalence of mobility impairment in the country is human immunodeficiency virus (HIV) and acquired immunodeficiency syndrome (AIDS). Myezwa et al. (2018) found that 39% of persons receiving antiretroviral therapy (ART) experience mobility limitations.

Rural areas mostly have poor infrastructure with long distances to travel in between service organisations (Vergunst et al. 2017; Visagie & Swart 2016). These factors mostly affect those with mobility impairments in accessing and using healthcare services.

Many existing studies on healthcare access have, however, looked generally at all persons with disabilities who are still using healthcare (Grut et al. 2012; Moodley & Rose 2015; Mutwali & Rose 2019; Vergunst et al. 2015, 2017; Visagie 2015) and did not specifically sample by impairment type and by those who were using public healthcare and stopped accessing public care intentionally.

Understanding the experiences of those who stopped accessing healthcare, particularly in the most vulnerable rural communities, is thus important. Additionally, understanding specific challenges related to impairment type is equally important because of the diverse needs that persons with disabilities face, even those with the same impairment type. This study explored experiences of those who stopped accessing healthcare, more specifically those with mobility impairments in a rural context. Mobility impairment refers to a loss of functioning in lower limbs or the loss of lower limbs as a result of a trauma, congenital or acquired conditions (Gudwana 2019). Mobility impairments can influence access to and use of services. This study explored the experiences of persons with mobility impairments in accessing healthcare and the reasons for nonuse of healthcare in Cofimvaba, Eastern Cape province, South Africa.

### Research design

A qualitative methodology was followed, using a descriptive approach to explore the experiences of persons with mobility impairments who had stopped accessing healthcare before December 2018. The major purpose of descriptive research is description of the situation as it exists at present (Kothari 2004). In a descriptive approach, a researcher obtains information of what happened. In this instance, the reasons for nonuse of healthcare and the challenges experienced were described.

### Setting

Cofimvaba is a poor, deeply rural area, situated in the Chris Hani District. It is 80 km away from Queenstown, the nearest town, in the Eastern Cape province. Cofimvaba forms part of the Intsika Yethu Municipality, which consists of 21 wards. It has 35 clinics, one community health centre (CHC) and one district hospital. Some villages are more than 20 km away from the clinics with bad roads, especially gravel roads with potholes and stones, which become muddy when it rains. In these areas, accessing public transport is a challenge. As a result, it is difficult to access services, as the natural environment on its own presents many mobility challenges for those with disabilities. According to the District Health Information System (DHIS), the total population of Cofimvaba is 135 933.

The first author chose this particular setting and study focus because she has been practising as an occupational therapist and rehabilitation manager in Cofimvaba Hospital for 13 years and had noticed, over the years, how persons with mobility impairments were disappearing from the system and no longer coming for follow-up check-ups, even for assistive devices. The first author was therefore interested in exploring the reasons for the nonuse of services.

### Study population and sampling strategy

The study population were all persons with mobility impairments in Cofimvaba who had stopped accessing formal public healthcare before 2018. This research focused on those who did not access healthcare for at least 12 months. Snowball sampling was used in this study, which involved building a sample through referrals (O’Leary 2017). Referrals were received from the community health workers who identified participants from the respective communities they serve.

Participants were selected from different communal wards. Initially, the plan was to conduct the interviews face-to-face; however, because of the coronavirus disease 2019 (COVID-19) pandemic, interviews were conducted telephonically. Having to conduct telephonic interviews also decreased the number of participants, as only five of nine were contacted on cell phone calls made on three different occasions. The information on the participant’s last date of visit to the facility was received from the community healthcare workers and then confirmed during the telephone interview with the participant.

### Inclusion and exclusion

The persons with mobility impairments who had stopped accessing formal, public healthcare before December 2018 were included in the study. Participants had to be 18 or older and able to express themselves in isiXhosa or English. Persons with impairments other than mobility were excluded from the study. The study participants who were able to express themselves in isiXhosa and English were included. Those with impairments other than mobility impairments or with a combination of mobility and other impairments, and participants with cognitive impairments, were excluded (see Table 1). Persons with other impairments were excluded because this study focused on mobility impairments.

**TABLE 1:** Demographic details of participants.

No. of participants sampled	Gender	Ward	Socio-economic status	Age	Type of impairment
P001	Male	19	Employed at Intsika Yethu Municipality	33	Polio and wheelchair user
P002	Male	18	Unemployed; dependent on disability grant	56	Paraplegia because of MVA and wheelchair user
P003	Male	3	Unemployed; dependent on disability grant	77	Bilateral amputee
P004	Female	20	Unemployed; dependent on disability grant	53	Lower limb weakness because of CVA and wheelchair user
P005	Male	20	Unemployed; dependent on disability grant	34	Bilateral amputee

MVA, motor vehicle accident; CVA, cerebrovascular accident.

### Data collection

Semistructured interviews were conducted to gather descriptive accounts of people’s experiences. Semistructured interviews are flexible and allow the discovering of individual responses regarding a particular situation or phenomenon (McIntosh & Morse 2015). In semistructured interviews, participants are free to respond to open-ended questions as they wish, and the researcher probes the responses for any further elaboration needed. The interviews were conducted in isiXhosa, using a self-developed interview guide. The self-developed interview guide had brief questions to start the conversation and enabled participants to express their experiences on accessing healthcare and reasons for stopping accessing healthcare. The first author used the self-developed interview guide to ensure that the tool provided the necessary information for which it was intended. It was divided into demographic and interview questions. The main questions were as follows:

- Tell me how it is for you to use healthcare.
- When was the last time you accessed healthcare?
- What are the reasons for stopping accessing healthcare?
- What challenges did you experienced while accessing healthcare?
- What are your feelings on accessing healthcare?
- What can be done so that you can access healthcare again?
- Anything you would like to add?

The tool was translated from English to isiXhosa by the first author and cross-checked by the third author. There was no back-translation. This is because the first and third authors are both fluent in isiXhosa and did not need to outsource a translator. Transcripts were transcribed and translated from isiXhosa to English. The tool was tested through a pilot study with one participant to ensure that questions were clear and to enable the researcher to practise doing an interview. There were a few changes that were made after the pilot study, such as improving the way the first author conducted the interview, asked for clarity and conducted probing. The pilot participant’s data was not part of the five participants cited in this study.

The interviews lasted between 30 and 45 min. There were problems that were encountered, such as network challenges which interfered with the quality of the interviews. Some participants were not reached at the agreed time because of signal issues. During the interviews, signal was at times lost, flat batteries occurred and faulty speakers of the phone made it difficult to hear what the participants were saying. As such, the first author had to request participants to repeat their responses often and also had to repeat questions to participants. At times, interviews were rescheduled for another day. These were all limitations that came with using virtual methods as a result of COVID-19 restrictions in rural contexts.

All interviews were recorded using an audio recording device, and the first author kept additional notes on interviews using a reflective journal. Each participant was assigned a code (alphanumerical, e.g. POO1) and they were not identified by their real names. All data were transcribed verbatim by the first author and then translated into English. Electronic data were kept in a password-protected folder on a desktop computer. The audiotapes and hard copies of transcriptions were kept in a locked cabinet in the first author’s office. Backup data were also stored in a password-protected cloud folder. All raw data will be destroyed after 5 years, as stipulated by the university rules.

Data lost during interviews were recovered during follow-up when participants were called for follow-up to give them an opportunity to add or correct where they may have been misrepresented. In these follow-up interviews, the first author read the transcript to the participants, and they were asked to correct any misinterpretations. Each participant was remunerated with a R100.00 airtime voucher for his or her network, such as Vodacom, MTN and the like, to thank them for the time they spent during the interview.

### Data analysis

Inductive thematic analysis, a bottom-up approach was used to analyse data (Braun & Clarke 2006). The researcher followed the six phases to thematic analysis, including familiarising self with the data, generating initial codes, searching for themes, reviewing themes, defining and naming themes and producing a final report (Braun & Clarke 2006, 2012). Identified codes were grouped together to develop and define themes. Recurring themes were grouped together to eventually form seven main themes, as set out in the results section.

### Ethical considerations

Permission was obtained from and approved by the Health Research Ethics Committee at Stellenbosch University (reference number S19/10/263). Participation in the study was voluntary. Participants gave verbal consent because of COVID-19 and later signed written informed consent forms which were translated to isiXhosa. Participants were informed that the study was independent; thus, no direct benefit would be derived from participation. Confidentiality was maintained throughout the entire research study between the researcher and the participants. All study participants were treated equally, thus ensuring fairness. Researcher explained to participants that the information would be kept anonymous and pseudonyms would be used in the writing up of the study findings.

### Trustworthiness

In this study, trustworthiness was enhanced by ensuring credibility, transferability, dependability and confirmability (Lincoln & Guba 1985). For credibility, the first author followed up with all participants to check that the transcripts accurately reflected what they shared. This was carried out after transcription. The second author also cross-checked the translated data against the isiXhosa data to ensure that meaning was not lost through translation. Raw data were presented in quotes. It cannot be assumed that the research findings will be transferable from one context to another. However, the authors provided rich contextual descriptions of the study and the methodological steps followed to enable transferability. A description of how codes were developed, how themes were identified and how conclusions and findings were reached was documented to address confirmability. Records of raw data, field notes and transcripts were also kept. A reflective journal was also used to document the first author’s emotions and experiences. To ensure dependability, the authors guarded against vagueness by making clear all the processes engaged in carrying out the study so that other researchers will be able to replicate the processes and achieve similar results.

## Results

Six themes emerged from participants’ experiences in accessing healthcare. These included inaccessible roads, geographic inaccessibility, financial inaccessibility or indirect cost of care, little or not many health problems, physical infrastructure difficulties within facilities and attitudinal barriers.

### Theme one: Inaccessible roads

Inaccessible roads were a cause of nonuse of health facilities for study participants. Inaccessible roads included unmaintained gravel roads and unpaved roads, sometimes with hilly terrain, mud and stones. These all made it difficult to use a wheelchair:

‘The road is gravel with stones, has holes and when it is raining, the road is muddy. I had difficulties to push wheelchair. My child used to assist me by pushing me in the bad road especially in the holes. I felt bad because the road has stones. My child, sometime he was at school and I had stress because the road is small, I was afraid what if a car might come I would in a problem.’ (P004, female, 53 years old)

Participants described that roads are not well-maintained:

‘The road is a gravel, its bad and not maintained. It is having potholes, holes, and the taxi driver will leave you next to those potholes. I struggled to push a wheelchair through those potholes. Taxi drivers do not have patience, they left me where ever they wanted to leave because they rushed to town. I ended up struggling to cross the road and the person that helped to push me also struggled because the road has stones and when it’s raining, there is mud that makes it difficult to push a wheelchair.’ (P005, male, 34 years old)

‘The road is muddy when it rains and cars don’t enter the village when it’s raining. There are rocks.’ (P003, male, 77 years old)

‘When it rains, I would get my arms and clothes dirty because I had to help the person who was pushing me. Sometimes I needed to be fetched because my wheelchair was not moving in mud. After I pushed myself in the mud I didn’t feel well, I had to sleep and rest, my body ached.’ (P002, male, 56 years old)

### Theme two: Geographic accessibility

A spatial factor felt by participants in these villages was the long distance between their villages and the nearest health facility. This was identified as a major challenge that led participants to stop using the health facility:

‘Clinic is far from the community more especially for persons with disabilities who are wheelchair bound. Most of transport/taxis from town are full, normally pass through while I was waiting at the bus stop and it is a problem because I ended up feeling sorry for myself. When you are a wheelchair bound person people get annoyed, irritated because I needed to be assisted in order to get inside the taxi.’ (P001, male, 33 years old)

‘If I was educated I will be able to measure the distance but all I can say it is too far from the community to the clinic as a result I cannot go by myself that’s why I had to hire a car but able bodied can walk.’ (P003, male, 77 years old)

‘It is far, from the home to the bus stop is also far, then I had to take a taxi to the clinic. Other people had to get to the taxi and the taxi went to the clinic. When I arrived at the clinic it’s around nine in the morning and I had to wait to be served by the nurse.’ (P002, male, 56 years old)

These distances need one to have transport. However, these participants experienced transport challenges as well, which exacerbated their access issues, and thus they resorted to no longer using the health facility. Their most common means of going to the clinic was through public taxis, but these are similarly difficult to access:

‘I had transport challenges most of the time especially when I had to go back home. To go to the clinic I did not have a challenge, most drivers understood me and accommodated me but the challenge was when I was supposed to go back home because taxis from our community during the day were in town. I sat next to the road and I ended up pushing my wheelchair and rested along the road. I felt bad end up asking yourself why God made men to be disabled using a wheelchair. I felt like a burden in most of people. Sometimes I don’t blame the drivers because the car manufacturers do not accommodate persons with disabilities when they design cars because there is no space for a wheelchair in the taxi. I ended up having difficulties in accessing healthcare.’ (P001, male, 33 years old)

‘Since I’m using a wheelchair I experienced problems with transport. I used to call a driver to spare a front seat and sometimes I did not have airtime. When I arrived at the taxi, the front seat was occupied and I used to request the persons in front so that I can sit in front and some of them refused and had to stop going to the clinic because I could not sit at the back of the van.’ (P005, male, 34 years old)

### Theme three: Financial accessibility and indirect cost of care

Public healthcare services are free for persons with disabilities. However, participants reported that they incurred costs to pay taxis in order to access the health facility. These costs sometimes escalate for persons with disabilities because they have to hire a taxi from home to the health facility:

‘When I went to the clinic I paid R150 and to town I paid R700 but when I went to the doctor I called a car that fetched me at my house and charged me R1000.’ (P003, male, 77 years old)

‘It depends when I hired a car I didn’t pay less than R500 from Mcambalala to Cofimvaba hospital.’ (P005, male, 34 years old)

‘A driver normally charged me R200 because I took him from his home but some drivers felt sorry for me and charged R30.’ (P004, female, 53 years old)

Some participants are dependent on a disability grant, and having to pay for a wheelchair in a taxi takes a good deal from the grant, among other competing needs for survival:

‘It is too much money, in other taxis I had to pay for myself a seat and a wheelchair and that made me feel bad because I’m only dependent on disability grant which is not sufficient to cater for my family needs.’ (P001, male, 33 years old)

Responding to referrals from the clinic to the hospital for further treatment similarly adds a cost to the participants as they had to find their own way to the hospital and these are far apart:

‘To go to Ncora clinic its R600, may be at Ncora they refer me to Cofimvaba hospital which is R900 … Since I’m getting a disability grant, I had challenges with buying grocery and my medication when I had to cover transport fees. Some medication is not available at the clinic especially those they gave me energy [*vitamins*]. I bought them from the chemist.’ (P002, male, 56 years old)

### Theme four: Little or not many health problems

Some participants in this study stated that not having any illnesses for a while stopped them from accessing the health facility for treatment:

‘No it’s been a long time not using a health clinic. There are no reasons but I hardly get sick and have nothing that needs me to come to the clinic. I don’t have another reason. I will visit the health clinic when I have illness.’ (P001, male, 33 years old)

‘If nothing is bothering you I don’t see the need for you to go to the clinic. Nothing forced me to go to the clinic. I rarely get sick, nothing would encourage me to go to the clinic. Without illness I don’t see if I can look for opportunities to go to the clinic. I’m still young to go to the clinic, I will attend the clinic when I’m old at 60 years. I will visit clinic when I’m sick.’ (P005, male, 34 years old)

Another 53-year-old woman in Upper Mncuncuzo described herself as a nonuser of health services because she had not had any health problems over the past 4 years:

‘I don’t have anything that needs me to go to healthcare. It’s been 4 years not attending healthcare. I attended healthcare a long time ago because I had pains when I was starting to use a wheelchair. I hardly get sick now but I will visit when I am sick.’ (P004, female, 53 years old)

### Theme five: Physical infrastructure difficulties within facilities

Some health facilities are not accessible to persons with mobility impairments because of inaccessible infrastructure. Participants described the toilets as inaccessible as they are not designed for persons with mobility impairments:

‘I normally don’t go to the toilet because I got helped but one day I wanted to relieve myself and I could not get inside because the toilet is outside, not designed for persons with disabilities, no handrails; it was difficult for me to use the toilet.’ (P002, male, 56 years old)

One participant had to crawl to get inside the toilet, which is a publicly dehumanising experience to persons with disabilities.

‘Toilets at clinic are not catered for persons with mobility impairment. They are outside, the door is too small, a wheelchair cannot get inside and you have to crawl to get inside the toilet. I was helped with no problems except the toilet problem.’ (P001, male, 33 years old)

Only one participant did not have challenges with inaccessible toilets:

‘The last time I visited hospital I used toilets and they were big enough for a wheelchair to get inside, with rails, clean and designed for persons with disabilities.’ (P005, male, 34 years old)

### Theme six: Attitudinal barriers

Prevalent negative attitudes of health professionals and attitudes of public taxi drivers were reported as one of the reasons that made using services difficult and thus made them stop accessing healthcare:

‘There is an attitude at the taxis towards wheelchairs that cannot get to the boot. The other thing they had to pick me from the wheelchair to the taxi they feel that it’s too much, wasting their time. That 10 minutes that they had to pick me to the taxi, rushing to another place so end up leaving me. I feel bad end up asking myself why God made me to be disabled using a wheelchair. I felt like a burden in most of people. Sometimes I don’t blame the drivers because the car manufacturers do not accommodate persons with disabilities when they design care because there is no space for a wheelchair in the taxi. I ended up having difficulties in accessing healthcare.’ (P001, male, 33 years old)

‘Some of the drivers passed through and said it’s full whereas it’s not full because I’m a burden that needs to be assisted to get inside the car and that makes me feel bad.’ (P004, female, 53 years old)

‘Using healthcare is well but there are challenges especially in some clinics here in the Eastern Cape. There seems to be some misunderstandings from the nurses for example when a disabled person gets to the clinic, pregnant, you find that nurses will question each other about how and why she is pregnant when she is disabled and who would impregnate a disabled person. There are also government condoms which are freely available. Sometimes when a person with a disability requests such condoms from the public health facility, there tends to be some hesitancy from nurses, wondering what you are asking the condoms for. Then they will say you like things.’ (P001, male, 33 years old)

## Discussion

This study reveals geographic inaccessibility as introducing many challenging experiences to persons with mobility impairments, including hindering access to essential services such as healthcare. The issue of long distance is prominently featured as a reason for nonuse of healthcare facilities, as well as poor access in other healthcare access studies for persons with disabilities. For example, long distances as a barrier to access are seemingly common in other rural contexts, including other parts of the Eastern Cape in South Africa (Vergunst 2016), in rural Namibia (Van Rooy et al. 2012), rural areas in South Asia (Gudlavalleti 2018), in Botswana (Paulus-Mokgachane, Visagie & Mji 2019), in Ghana (Appiah et al. 2020), in Malawi (Munthali et al. 2019) and other rural areas in LMICs (Dassah et al. 2018). In all these studies, long distance to a healthcare facility is reported as a major barrier to accessing healthcare for persons with disabilities. Interestingly, some urban areas have also revealed the same. For example, Scheffler, Visagie and Schneider (2015) identified that most users with disabilities in urban Cape Town access health facilities by foot as they are within a 3 km radius. De Klerk et al. (2019) in Cape Town also recently identified that travelling distance is attributed to not attending physiotherapy and occupational therapy appointments. However, what worsens access challenges in more rural contexts is the finding that even for those in close proximity, more problems arise including poor terrain, gravel and muddy, poorly maintained roads with rocks which make it difficult to access healthcare when one has mobility impairments. This is often worse after heavy rains, as navigation becomes difficult and challenging for wheelchair users to propel themselves, as the participants in this study revealed. The rural landscape is replete with examples of spaces that explicitly segregate persons with disabilities into differing spatial spheres, that is, inaccessible toilets, roads with no wheelchair-friendly ramps, places and services linked by inaccessible public transport and so on. This is because many of these have not been designed and built with persons with disabilities in mind. One may argue that the generally communicated message is that they are ‘out of place’.

Another theme that needs to be addressed is the process of travelling to the clinics. The experiences necessitate good access to transport, whether private or public. A majority of the South African population rely on public transportation, and this is limited in many rural settings.

This study shows participants who experienced transport challenges ranging from unavailability to unaffordable and/or inaccessible transport. Compounding this was the challenge of being sometimes met with negative attitudes from taxi drivers or even long waiting hours because of some taxis not wanting to take a person with a disability. All these contributed to their nonuse of healthcare services. Similarly, Magnusson, Finye and Enstedt (2020) in Malawi recently reported unavailability of transport as the reason for prosthetic and orthotic users not attending healthcare, and Vergunst et al. (2016) also found the lack of transport to healthcare facilities as a hindrance to health access in another rural area in the Eastern Cape, South Africa.

While others could manage hiring private taxis at times, this was, however, not a sustainable solution, as it became too expensive. It always became too costly to pay the extra fare to cover the wheelchair space or that of the personal assistance (Tshaka 2021). Previous studies have well documented the additional costs that persons with mobility impairments incur when accessing healthcare (Eide et al. 2015; Hashemi et al. 2020). This is to say, transportation is one of the factors that introduces added costs and is a barrier to accessing health services.

When persons with mobility impairments finally arrive at these facilities, they are also presented with other challenges, such as inaccessible toilets in their respective clinics and facing negative attitudes from health professionals. Negative attitudes from health professionals are a longstanding concerning issue to persons with disabilities (Eide et al. 2015; Munthali et al. 2019; Ntamo, Buso & Longo-Mbenza 2013; Van Rooy et al. 2012). This is similar for toilets which are not designed for inclusiveness – a violation of the human rights of persons with disabilities which makes visits to health centres not a pleasant experience. For example, Hashemi et al. (2020) reported that persons with disabilities often avoid drinking liquids when accessing healthcare centres because toilets are inaccessible.

Health and planning are historically linked. A notable feature in South Africa is that the geographic accessibility challenges experienced by persons with disabilities remain a symptom of the colonial and apartheid administration which created not only interprovincial differences in access but also rural and urban differences and inequities in accessing services – differences which led to the underdevelopment of rural areas and services. This study shows that there is an umbilical link between environmental conditions in rural contexts, healthcare access challenges and the nonuse status. It is important to recognise more fully the impact these environmental factors have on human emotional and stress responses which lead them to stop using services that they need. This may have poor impact on the health outcomes of persons with disabilities, if not addressed.

## Conclusion

Having clinics and hospitals in the community is of little use if one cannot travel to them because of long distances, poor infrastructure and inaccessible transport. It is clear that issues of accessibility are much more complex than having services located closer. Persons with mobility impairments as a group have reduced mobility and live in relative poverty, which restricts their ability to own and run a car. As a consequence, they are reliant on public transport and taxis to travel, with assistance from friends and family. However, these resources are not always available at convenient times. This is exacerbated in rural settings where public transport is largely inaccessible and where people struggle with financial accessibility too. Additionally, family and friends are also sometimes at work or school and thus unable to provide the needed support of accompanying a relative to the clinic. A solution, for example, might be for local authorities to provide transport on demand until the public transport system becomes more inclusive. Alternatively, investing in community-based workers who are trained on working with persons with disabilities could be a form of support. Additionally, efforts need to be directed towards fixing rural roads infrastructure so that persons with mobility impairments can navigate them with ease. Lastly, the local Department of Health must come up with strategies on eliminating negative attitudes of health professionals towards persons with disabilities, as this impedes access to services.

### Limitations

Cofimvaba is a vast, deeply rural area with hard-to-reach areas because of the poor condition of roads. As a result of the COVID-19 pandemic, verbal consent was gained as participants did not have e-mail addresses and the postal service would have imposed further delays. Later, however, written consent forms were sent to participants via community health workers for signature and filling. Because of poor connectivity and limited access to electricity in rural areas, network connectivity challenges in some areas imposed difficulties to reach some participants telephonically. Some participants experienced challenges with their telephones, for example, battery life time, faulty speaker, poor connection or last-minute challenges such as family and health problems. The small sample is also a limitation to this study, as these findings cannot be generalised. It should, however, be noted that the aim of qualitative research is not to be generalisable but to provide rich data, which was achieved in this study.
